# Brain and behavioral lateralization in invertebrates

**DOI:** 10.3389/fpsyg.2013.00939

**Published:** 2013-12-11

**Authors:** Elisa Frasnelli

**Affiliations:** Center for Mind/Brain Sciences, University of TrentoRovereto, Italy

**Keywords:** brain and behavioral lateralization, invertebrates, individual efficiency, directional asymmetry, evolutionary stable strategy, bee, sociality

## Abstract

Traditionally, only humans were thought to exhibit brain and behavioral asymmetries, but several studies have revealed that most vertebrates are also lateralized. Recently, evidence of left–right asymmetries in invertebrates has begun to emerge, suggesting that lateralization of the nervous system may be a feature of simpler brains as well as more complex ones. Here I present some examples in invertebrates of sensory and motor asymmetries, as well as asymmetries in the nervous system. I illustrate two cases where an asymmetric brain is crucial for the development of some cognitive abilities. The first case is the nematode *Caenorhabditis elegans*, which has asymmetric odor sensory neurons and taste perception neurons. In this worm left/right asymmetries are responsible for the sensing of a substantial number of salt ions, and lateralized responses to salt allow the worm to discriminate between distinct salt ions. The second case is the fruit fly *Drosophila melanogaster*, where the presence of asymmetry in a particular structure of the brain is important in the formation or retrieval of long-term memory. Moreover, I distinguish two distinct patterns of lateralization that occur in both vertebrates and invertebrates: individual-level and population-level lateralization. Theoretical models on the evolution of lateralization suggest that the alignment of lateralization at the population level may have evolved as an evolutionary stable strategy in which individually asymmetrical organisms must coordinate their behavior with that of other asymmetrical organisms. This implies that lateralization at the population-level is more likely to have evolved in social rather than in solitary species. I evaluate this new hypothesis with a specific focus on insects showing different level of sociality. In particular, I present a series of studies on antennal asymmetries in honeybees and other related species of bees, showing how insects may be extremely useful to test the evolutionary hypothesis.

## INTRODUCTION

Until some decades ago, it was widely and incorrectly assumed that lateralization of structure and behavior was unique to the human brain, and having a lateralized brain was a mark of the cognitive superiority of humans. Now it is well known that most vertebrates have strong left–right asymmetries in their brain and in their behavior and lateralization is widespread in the vertebrate subphylum (for a review on handedness, see Ströckens et al., 2013; for a review on language lateralization, see Ocklenburg et al., 2013). Moreover, lateralization has a similar plan of organization in different species (for a review, see Rogers et al., 2013a). Recently, new evidence has shown the presence of lateralization in invertebrate species, suggesting that lateralization of the nervous system may be a feature of simpler brains as well as more complex ones (for a fully comprehensive review, see Frasnelli et al., 2012a). Some invertebrates show a lateralized behavior in motor control, other species exhibit asymmetries in several sensory modalities, such as in olfaction or vision, and in some cases behavioral lateralization seems to be correlated with a morphological one. In this section I present briefly some examples. In the second section I focus on two examples of brain asymmetries in invertebrates – fruit fly and nematode – that show how lateralization at the individual level is important to perform specific cognitive abilities. Then, in the third section, I explain that two patterns of lateralization exist, i.e., individual level and population level lateralization, I discuss how the latter may have evolved as an evolutionary stable strategy (ESS) and I focus on insects to provide evidence to test the ESS hypothesis. Finally, in the forth and last section, I conclude by comparing lateralization in invertebrates and vertebrates and discussing its possible evolutionary origins.

### MOTOR ASYMMETRIES

Ants (*Formicidae*) and spiders (*Araneae*) were found to be lateralized (Heuts et al., 2003). A significant majority of spiders were observed to have mainly left leg lesions, and the process of catching them caused less severe leg lesions that were also significantly biased to the left. Similarly, Ades and Ramires (2002) showed that the spitting spider *Scytodes globula *(Arachnida, Araneae, Scytodidae) uses its left anterior legs considerably more frequently than the right anterior legs during prey handling. Twelve ant species of *Lasius niger* kept mainly to the right on their foraging “streets,” whereas there was only one species that kept to the left (Heuts et al., 2003).

Behavior of the common American cockroach, *Periplaneta americana *(Linnaeus) has been investigated to determine whether lateralization is evident in a bias to turn left or right (Cooper et al., 2011). The cockroaches were allowed to run through a Y-tube and make a choice of which direction to take. Vanilla and ethanol were placed randomly at the ends of the Y-tube to entice the cockroaches to reach the end of the tubes. Thirty-eight adult cockroaches were tested for each of the following five conditions: both antennae intact, half of the left antenna cut, all of the left antenna cut, half of the right antenna cut, and all of the right antenna cut. Results showed that the odors of vanilla and ethanol play an insignificant role in the decision-making. Injury of one antenna affected the choice of direction, but not in a consistent way. While the majority of cockroaches with an amputated left antenna chose to go right, this did not happen when the entire right antenna was removed. In fact, similar injuries to either the right or the left antenna revealed an innate bias for turning right. Similar results were obtained when either antenna was cut in half. More evident was the skew towards the right path when both antennae were intact. The antennae of these gregarious insects are very long and, in addition to their role in detecting chemicals, they are very important as tactile organs (Okada and Toh, 2004). The study by Cooper et al. (2011) thus suggests that *Periplaneta americana *has a motor bias towards the right and not that this species is right-side dominant in its tactile and odor senses. Cockroaches turn right when there is no sensory input from the antennae, showing that they have a motor bias, and input from the antennae modifies this motor bias, often to reduce its strength.

Evidence of lateralized behavior has been found in the giant water bugs, *Belostoma flumineum Say* (Heteroptera: Belostomatidae; Kight et al., 2008). Giant water bugs are large aquatic insects, predators of other aquatic invertebrates, and small fishes. Bugs were trained to swim left or right in a T-maze and a significant preference to turn left, even when not reinforced, was observed, revealing a naïve bias in this species. To control for environmental cues that might bias the turning direction of water bugs in the maze, Kight et al. (2008) ran two separate experiments on independent groups of 20 water bugs. Both experiments were identical with the exception that, after the first group of 20 water bugs had been tested, the maze apparatus was rotated 180°, thereby reversing the polarity of all directional environmental cues such as lighting or electromagnetic fields. Again the same left turn tendency was observed. Hence, the explanation of the presence of this bias could be the existence of asymmetries in the nervous system or asymmetric exoskeletal morphology (i.e., leg length) that could cause biased swimming behavior.

### PERCEPTUAL ASYMMETRIES

Fruit flies *Drosophila melanogaster* present a consistent asymmetry in the antenna-mediated flight control, in which the sensory signals coming from the left antenna contribute more to odor tracking than the sensory signals coming from the right antenna (Duistermars et al., 2009). The rapid odor lateralization in *Drosophila* is enabled by an asymmetric neurotransmitter release (Gaudry et al., 2013): each olfactory receptor neuron (ORN) spike releases ~40% more neurotransmitter from the axon branch ipsilateral to the soma, as compared to the contralateral branch. This implies that, when an odor activates the antennae asymmetrically, ipsilateral central neurons begin to spike a few milliseconds before contralateral neurons, and ipsilateral central neurons also fire at a 30–50% higher rate. As a consequence, a walking fly can detect a 5% asymmetry in total ORN input to its left and right antennal lobes, and can turn toward the odor in less time than it requires the fly to complete a stride (Gaudry et al., 2013).

Red wood ants *Formica aquilonia* were found to use mainly their right antenna during “feeding” contacts where a “donor” ant exchanges food with a “receiver” ant through trophallaxis (Frasnelli et al., 2012b). Honeybees *Apis mellifera* seemed to use primarily their right eye for learning to associate a visual stimulus with a food reward (Letzkus et al., 2007).

Individual octopuses have significant eye preference for viewing a crab held outside the tank, but there is no population-level bias (Byrne et al., 2002, 2004).

An asymmetry in T-maze behavior has been reported in the cuttlefish *Sepia officinalis* trained to learn how to enter a dark, sandy compartment at the end of one arm of the maze (Alves et al., 2007). Eleven out of 15 cuttlefish displayed a pervasive side-turning preference. A further study by Alves et al. (2009) on a large sample (*N* = 107), confirmed the existence of a population-level bias. To find out whether or not visual perception plays a role in determining the direction of turning, cuttlefish were tested either inside the empty apparatus or with attractive visual stimuli (sand and shadow) on either sides of the T-maze. The authors (Alves et al., 2009) found that in both cases there was a preference to escape leftwards and they suggested that this left-turning bias results from an eye use preference. This visual lateralization observed in cuttlefish is task and age dependent (Jozet-Alves et al., 2012a). Cuttlefish were tested in a T-maze during postembryonic development (3, 7, 15, 30, and 45 days) in two different configurations of the apparatus, i.e., by providing or not shelters in the two choice arms of the maze to determine whether or not the direction of turning was stimulus dependent. Cuttlefish developed a left-turning bias from 3 to 45 days post-hatch (no bias at 3 or 7 days, bias at 15, 30, and 45 days) but only when shelters were provided in the apparatus (Jozet-Alves et al., 2012a). The left-turning bias is associated with a right visual hemi-field and thus a right eye preference. Cerebral correlates of this visual lateralization have been found by looking at anatomical (vertical lobe – VL, peduncle lobe – PL, inferior buccal and optical lobe – OL; Nixon and Young, 2003) and neurochemical (monoamines in OL) brain asymmetries and at their correlation with behavior (Jozet-Alves et al., 2012b) in cuttlefish at 3 and 30 days post-hatching. Brain and behavior asymmetries were present only at 30 days post hatching: a population level bias towards a larger PL and higher monoamine concentration (i.e., serotonin, dopamine, and noradrenaline) in the left OL was observed (Jozet-Alves et al., 2012b). Interestingly, there was a correlation with the behavioral results in the T-maze: the larger the right OL and the right part of the VL, the stronger the bias to turn leftwards. Jozet-Alves et al. (2012b) also observed one individual with the left OL larger and a bias to turn rightwards, which is evidence of a minority of cuttlefish lateralized in the opposite direction. Embryonic exposure to predator odor modulates visual lateralization (Jozet-Alves and Hébert, 2013). A left-turning bias in T-maze for cuttlefish exposed to predator odor (sea-bass) prior to hatching was observed; whereas no bias for embryos exposed to non-predator odor (sea urchins) or for those incubated with no odor (blank tank) was found. Moreover, when tested with predator odor in the apparatus all cuttlefish display a left-turning preference, suggesting an ability to innately recognize predator odor (Jozet-Alves and Hébert, 2013).

In the deep-sea squid *Histioteuthis* the left eye and the left optic lobes are considerably larger than their equivalents on the right side (Wentworth and Muntz, 1989). The left eye appears to be used to look upwards into the better-lit upper waters, possibly to detect predators. The smaller right eye looks downwards, perhaps searching for bioluminescence, probably prey. Male squid *Sepioteuthis* can give courtship color displays to a female on one side, while giving a threat display to a male on the other side (Messenger, 2001). Asymmetrical color display is also a characteristic in cuttlefish (Brown et al., 2012). Male mourning cuttlefish (*Sepia plangon*) deceive rival males by displaying male courtship patterns to receptive females on one side of the body, and simultaneously displaying female patterns to a single rival male on the other (Brown et al., 2012). This evidence in cephalopods shows a capacity for considerable independence of motivational control on the two side of the central nervous system, a capacity that confers advantages on the individual.

### FUNCTIONAL ASYMMETRIES

*Limax* slugs trained to avoid a particular food odor may hold the memory in either the right or the left procerebral division of the brain with the equal likelihood (Matsuo et al., 2010). However, when the right side is damaged by ablation, memory is fully affected, suggesting that learning and/or memory may be lateralized processes.

A behavioral asymmetry in mating behavior, due to an anatomical asymmetry dependent on a maternal effect gene, has been observed in the pond snail *Lymnaea stagnalis* (Asami et al., 2008; Davison et al., 2009). The pond snail *Lymnaea stagnalis* is a self-fertilizing hermaphrodite; in any single mating an individual takes the male role or the female role. Chirality in snails is determined by the single locus of the maternal effect (Boycott and Diver, 1923), i.e., the phenotype of an individual is dependent upon the genotype of their mother. Asami et al. (2008) used crossing experiments to demonstrate that the primary asymmetry of *L. stagnalis* is determined by the maternal genotype at a single nuclear locus where the dextral allele is dominant over the sinistral allele. Dextral is dominant in *Lymnaea* (by convention D = dextral allele; S = sinistral allele). The dextral and sinistral stocks are genetically DD or SS, respectively. On mating virgin sinistral and dextral types, offspring (F1 generation) that are genetically dextral (genotype = DS) but with a shell coil that is either sinistral (sinistral mother) or dextral (dextral mother) are produced (F1 generation). By allowing the sinistral F1 mother to self-fertilize, offspring that have a dextral coil, but are genetically DD, DS, or SS are produced (F2 generation). Dextral SS individuals were identified by virtue of their producing sinistral young. Davison et al. (2009) investigated the occurrence and the inheritance of a potential laterality trait in the pond snail and tried to understand whether laterality traits are associated with both body chirality and nervous system asymmetry. They found that all dextral “male” snails, both those paired with dextral and those paired with sinistral, circled in a counter-clockwise manner. Similarly, all the sinistral snails circled in a clockwise manner, regardless of whether they were paired with another dextral or a sinistral snail. The circling direction of the sinistral male was independent of the chirality of the female. It was instead entirely dependent on the maternal genotype, rather than the individual’s own genotype.

Chirality in mating behavior is matched by an asymmetry in the brain. *L. stagnalis* has a ring of nine ganglia that form a central nervous system around the esophagus, with two more distant buccal ganglia on the buccal mass. In all dextral individuals, the right parietal ganglion is fused with the visceral ganglion and the left visceral ganglion is unpaired. By contrast, in all sinistral individuals the reverse is observed; the left parietal ganglion is formed by fusion with a visceral ganglion. The central nervous system in sinistral pond snails, therefore, has an asymmetry that is the reverse of that of dextral snails. As the coil of the shell is determined by the maternal chirality genotype and the asymmetry of the behavior is in accordance with this, it is likely that the same genetic locus, or a closely linked gene, determines the behavior. These findings suggest that the lateralized behavior of the snails is established early in development and is a direct consequence of the asymmetry of the body.

## THE ADVANTAGES OF HAVING AN ASYMMETRICAL BRAIN

Irrespectively of the kind of asymmetry, having an asymmetrical nervous system seems to give the individual some advantages. Lateralized animals have been shown to outperform non-lateralized in many circumstances (McGrew and Marchant, 1999; Güntürkün et al., 2000; Rogers et al., 2004), suggesting that lateralization contributes significantly to biological fitness. A lateralized brain may confer several advantages: sparing neural tissue by avoiding duplication of functions in the two hemispheres (Levy, 1977); processing information in parallel (Rogers, 2002; Rogers et al., 2004); and preventing the simultaneous initiation of incompatible responses by allowing one hemisphere to have control over actions (especially in animals with laterally placed sensory organs, Andrew, 1991; Vallortigara, 2000). Moreover, Rogers (2000) suggested that enhanced cognitive ability is one of the potential benefits of cerebral lateralization because animals with strongly lateralized brains may have the ability to act directly on many sources of information at the same time. Lateralized individuals are better able to distinguish food grains from pebbles compared with non-lateralized individuals (Güntürkün et al., 2000), and this disparity is enhanced in the presence of predators (Rogers et al., 2004). Similarly, chimpanzees that fish for termites using one hand are more efficient than ambidextrous individuals (McGrew and Marchant, 1999). Recently, the influence of lateralization on problem solving by Australian parrots (eight species) has been examined (Magat and Brown, 2009). In both a pebble-seed discrimination test and in a string-pull problem, strongly lateralized individuals (those showing significant foot and eye biases) outperformed less strongly lateralized individuals, suggesting that cerebral lateralization conveys a significant foraging advantage and supporting the enhanced cognitive function hypothesis.

Interestingly, not only in vertebrates but also in invertebrates an asymmetric brain is crucial for the development of some cognitive abilities. Two examples of invertebrate species where brain asymmetry at the individual level can confer advantages to the individual and, moreover, is necessary for the animal to have some cognitive abilities, are provided by the fruit fly *D. melanogaster* and the nematode *Caenorhabditis elegans*.

### THE FRUIT FLY *DROSOPHILA MELANOGASTER*

In the fruit fly, a structure located near the fan-shaped body connects the right and the left hemispheres (Heisenberg, 1994). This structure is an asymmetrical round body (called AB) with a diameter of about 10 μm and is not characteristic of all flies, since some flies have symmetry in this region. In a sample of 2,550 wild-type flies, 92.4% of individuals were found to have the AB in the right side of the brain (Pascual et al., 2004). Wild-type flies presenting symmetric structures were trained to associate an odor with an electric shock: a single training cycle was used for short-term memory testing and five individual training sessions (15-min rest intervals) for long-term memory testing. Pascual et al. (2004) observed no evidence of 4-day long-term memory in wild-type flies with a symmetrical structure, although their short-term memory was intact. On the contrary, flies with the asymmetrical structure formed long-term memory. Thus, brain asymmetry is not necessary for the *Drosophila* to establish short-term memory but it is important in the formation or retrieval of long-term memory.

### THE NEMATODE *CAENORHABDITIS ELEGANS*

The second example concerns one of the smallest existing nervous systems, namely the nematode *C. elegans*. With its 302 neurons, the nematode offers a unique opportunity to address the manner in which symmetrical neuronal assemblies deviate to create functional lateralization. Hobert et al. (2002) have provided a detailed cellular and molecular description of left-right (L-R) asymmetry in the nervous system of *C. elegans*. In this species, 2/3 of the neurons (198 out of a total of 302) are present as bilaterally symmetrical pairs. Particularly intriguing components of L-R asymmetry in the *C. elegans* nervous systems are neuron pairs (or neuroblasts) that are bilaterally symmetrical in terms of their post-morphogenetic position, morphology and lineage, but at some point during embryogenesis, after bilaterality has been established, undergo L-R-specific sub-differentiation programs. This is the case of the Amphid Single-ciliated Endings, ASEL (left)/ASER (right) neurons that are the main taste receptors of *C. elegans*. ASEL and ASER are bilaterally symmetrical with regard to cell position, axon morphology, outgrowth and placement, dendritic morphology, and qualitative aspects of synaptic connectivity patterns. However, three putative sensory receptors of the guanylyl cyclase class, gcy-5, gcy-6, and gcy-7, are expressed asymmetrically in ASEL (gcy-6, gcy-7) and ASER (gcy-5), two to left and one to the right (Yu et al., 1997). This asymmetry of gene expression correlates with a significant functional asymmetry of the two neurons: laser-ablation studies revealed that each of the individual neurons is responsible for sensing a distinct class of water-soluble chemicals (Pierce-Shimomura et al., 2001). Ortiz et al. (2009) investigated the extent of functional lateralization of the ASE neurons and genes responsible for the left/right asymmetric activity of ASEL and ASER. They showed that a substantial number of salt ions are sensed in a left/right asymmetric manner and that lateralized responses to salt allow the worm to discriminate between distinct salt ions.

## LATERALIZATION AT THE INDIVIDUAL AND AT THE POPULATION-LEVEL

In the two examples above lateralization at the individual level is fundamental for the individual to be able to perform some specific cognitive abilities, such as long-term memory formation in the fruit fly or discrimination of salt-ions in the nematode. However, it is worth emphasizing that behavioral (and brain) left-right asymmetries usually occur not only in single individuals but also in the same direction in most individuals. In this case, where most individuals show a similar direction of bias the group or population is biased, and so we speak of population-level lateralization. Individual brain efficiency does not require a definite proportion of left- and right-lateralized individuals. Thus, the arguments about the fact that brain lateralization increases individual efficiency do not explain population-level lateralization. Moreover, lateralization at the population level can also present ecological disadvantages, because it makes individual behavior more predictable to other organisms, such as predators. Theoretical models on the evolution of lateralization (Ghirlanda and Vallortigara, 2004; Vallortigara, 2006; Ghirlanda et al., 2009) suggest that the alignment of lateralization at the population level may have evolved as an ESS in which individually asymmetrical organisms must coordinate their behavior with that of other asymmetrical organisms (Vallortigara and Rogers, 2005). The hypothesis of the ESS of lateralization makes the quite straightforward prediction that initially “social” organisms would have started to be lateralized at the population-level, whereas “solitary” organisms retained lateralization at the individual level only.

### INSECTS TO TEST THE EVOLUTIONARY STABLE STRATEGY THEORY

Invertebrates and in particular insects have been excellent models to test the hypothesis predicted by the theoretical models on the evolution of lateralization that directional (population-level) asymmetry should be found only in cooperative, social species (Anfora et al., 2010). In fact, insects are among the certain current-living species in which the distinction between solitary and gregarious behavior can be defined quite sharply with respect to at least some aspects of behavior and in which it is likely that no major changes in sociality have occurred in evolutionary terms. Thus, the comparison of lateralization in social and non-social insects may provide a powerful test for the theory (Ghirlanda and Vallortigara, 2004; Vallortigara and Rogers, 2005). In particular, among Hymenoptera closely related species have evolved either sophisticated eusociality or maintained solitary behavior.

### THE HONEYBEE *APIS MELLIFERA*

**FIGURE 1:**
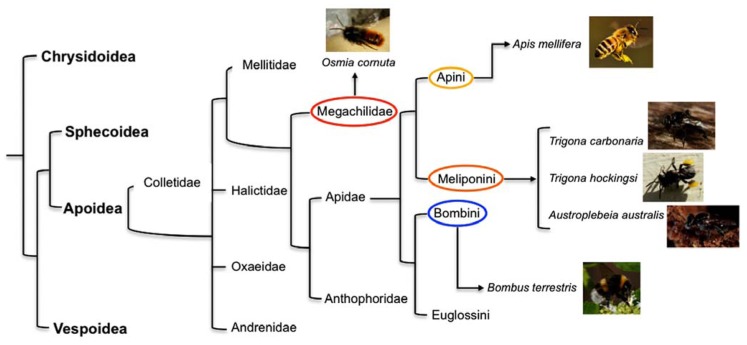
**A “family tree” for the Aculeata (hymenoptera with stings) showing the most likely relationship between super families (in Bold) and, for the super family Apoidea, the deduced lines of descent of some of the more common families of bees and tribes within the family Apidae ([Bibr B56])**. Highlighted are the tribes to which the species investigated for behavioral and brain asymmetries described in the current paper belong: in red the family Megachilidae (mason bee *Osmia cornuta*), in yellow Apini (honeybee *Apis mellifera*), in orange Meliponini (stingless bees *Trigona carbonaria*, *Trigona hockingsi*, *Austroplebeia australis*), and in blue Bombini (bumble bee *Bombus terrestris*).

Letzkus et al. (2006) first showed that honeybees *Apis mellifera* (Fam. Apidae, Tribe Apini – **Figure 1**) display laterality in learning to associate an odor with a sugar reward. The researchers used the proboscis extension reflex (PER) paradigm (Bitterman et al., 1983), in which bees are conditioned to extend their proboscis when they perceive a particular odor that has been associated with a food reward. They tested three groups of bees: the bees in one group had their left antenna covered with a silicone compound, which prevents detection of odor, those in the second group had their right antenna covered, and those in the third group constituted a control in which both antennae were uncovered. Bees with the right antenna covered learned less well than the bees with their left antenna covered and bees with both antenna uncovered. Frasnelli et al. (2010b) duplicated the behavioral results of Letzkus et al. (2006) using forager Italian honeybees (*Apis mellifera ligustica *Spin.) and checked for morphological differences in the number of sensilla between the right and the left antenna. Results showed that putative olfactory sensilla (*placodea, trichodea, basiconica*) were significantly more abundant on the right antenna surface than on the left antenna surface (mean difference of 3%), whereas sensilla not involved in olfaction (*campaniformia, coeloconica, chaetica*) were more abundant on the left than on the right antenna surface (mean difference of 7%). However, it seems unlikely that this can account for the functional asymmetry.

Rogers and Vallortigara (2008) investigated whether lateralization could be found in recall of olfactory memory at various times after the bees had been trained using the PER paradigm. At 1–2 h after training, using both antennae, recall of short-term memory was possible only when the bee used its right antenna but at 23–24 h after training the long-term memory could be recalled only when the left antenna was in use. Hence, retrieval of olfactory learning is a time-dependent process, involving lateralized circuits. Moreover, Rogers and Vallortigara (2008) also checked whether the laterality was manifested as side biases to odors presented to the left or right side of the bee without coating of the antennae. Bees were trained with both antennae in use and the recall was tested 1, 3, 6, or 23 h after using lateral presentation of the stimuli instead of coating the antennae. At 1 h after training, the correct responses were higher when the odors were presented on the right side than on the left side. At 3 h after training, no significant left-right difference was observed. At both 6 and 23 h after training the correct responses were higher when the odors were presented on the left side than on the right side.

Frasnelli et al. (2010a) tested lateralized recall of olfactory memory in honeybees at 1 or 6 h after training using different odors, including a familiar appetitive odor (rose) as a negative stimulus and a naturally aversive odor (isoamyl acetate, IAA – alarm pheromone) as a positive stimulus. The results confirmed the finding by Rogers and Vallortigara (2008). Moreover, it was found that the dynamic of memory traces has marked consequences when odors are already known to the bees (either for a biological reason or as a result of previous experience) and are thus already present in the long-term memory store. As a result, response competition arising from multiple memory traces can be observed, with bees showing unexpected lack of specificity in their longer-term olfactory memories.

A strong odor dependence of the lateralization of short-term memory recall of odors has been reported in honeybees (Rigosi et al., 2011). After training with 1-octanol and 2-octanone, bees showed no differences in the recall test regardless of whether they had use of only their right antenna, only their left antenna or both antennae. In contrast, bees trained with (-)-linalool showed a significant effect of the antenna in use: bees trained (and tested) with their right antenna in use performed significantly better than individuals with only their left antenna in use, whereas they performed the same as bees with both antennae in use (Rigosi et al., 2011). The odor (-)-linalool is one of the most common derivates of floral scents playing a crucial role as cue for pollinators (Knudsen et al., 1993). The odors 1-octanol and 2-octanone are unspecific and ubiquitous volatiles released from the green organs of the plants and thus of minor importance in pollinator plant interaction. Honeybees are able to learn complex odor mixtures by using a subset of key odors, such as (-)-linalool (Reinhardt et al., 2010) and, after conditioning bees to a mixture of odors (-)-linalool elicits higher levels of responding than do other components of the mixture presented singly (Laloi et al., 2000). Since bees are selective in their responses to odors, the strikingly different biological relevance of the odor compounds used by Rigosi et al. (2011) might be a reason for the observed difference in lateralization.

The asymmetry observed in the retrieval of olfactory learning in honeybee is much more complex than a difference in learning ability of the right and left antennae and the difference in number of olfactory sensilla is unlikely to explain entirely the behavioral laterality. Up to now, however, search for anatomical correlates of the asymmetry in higher centers of the bee brain has not revealed clear anatomical asymmetries (Haase et al., 2011a,b; Rigosi et al., 2011).

### Sociality and lateralization in Apoidea

It is important to underline that the studies mentioned above conducted on eusocial honeybees found an olfactory asymmetry in learning and recall of memory that manifests itself as population-level bias (i.e., the same pattern of lateralization was found in most individuals). Anfora et al. (2010) compared the behavior and electrophysiological lateralization of olfactory responses in two species of the superfamily Apoidea, the social honeybee, *Apis mellifera* L. (Fam. Apidae), and the solitary mason bee, *Osmia cornuta* (Latreille; Fam. Megachilidae – **Figure 1**). Unlike honeybees, mason bees are solitary: every female is fertile and makes its own separate nest, they don’t produce honey or wax and there are no workers (Nepi et al., 2005). Lateralization in mason and honeybees was tested using the PER paradigm. Bees were trained to associate an odor with a sugar reward and the recall of olfactory memory was tested at 1 h after training. The recall was better in honeybees when they used their right antenna than when they used their left antenna, confirming previous results obtained in the same species (Letzkus et al., 2006; Rogers and Vallortigara, 2008). Hence, honeybees show population-level lateralization. No such asymmetry was observed in mason bees. Consistent with this species difference, electroantennographic responses to a floral volatile compound and to an alarm pheromone were higher in the right that in the left antenna in honeybees but not in mason bees. Although the mason bees showed no population-level lateralization, they did show individual-level lateralization in that individual mason bees exhibited significant stronger responses either with the right or the left antenna, without any alignment of lateralization in the majority of the individuals. These data fit nicely with the hypothesis predicted by the theoretical models on the evolution of lateralization that links directional asymmetry with social behavior.

Olfactory asymmetries have been investigated also in bumblebees *Bombus terrestris *(Fam. Apidae, Tribe Bombini – **Figure 1**), an annual social species of bees. Anfora et al. (2011) ran a series of experiments similar to those conducted on mason and honeybees (Anfora et al., 2010). Bumblebees were trained to associate an odor with a reward using the PER paradigm and recall of memory was tested 1 h after. As for honeybees (Letzkus et al., 2006; Rogers and Vallortigara, 2008; Anfora et al., 2010; Frasnelli et al., 2010b), the bumblebees with the left antenna coated performed as well as those with both antennae in use, whereas bumblebees with the right antenna coated performed significantly less well. In contrast to honeybees, no significant differences were observed in electroantennographic responses between the left and right antennae of bumblebees when stimulated by two different compounds (an alarm pheromone and a floral scent). Interestingly, however, one class of bumblebee olfactory sensilla, *trichodea type A*, was shown to be more abundant on the surface of the right antenna than on the left one, and a slight tendency towards asymmetry was shown for a second class, i.e., *sensilla coeloconica*. Since electroantennographic responses represent the sum of responses of all ORNs housed in the sensilla of a single antenna (Schneider and Kaissling, 1957), the fact that morphological asymmetries were apparent only in a limited class of receptors may explain why, dissimilar to honeybees, no overall asymmetry was observed in EAG responses in bumblebees.

Kells and Goulson (2001) reported that bumblebees *Bombus* spp. show preferred directions of circling as they visit florets arranged in circles around a vertical inflorescence. In three (*Bombus lapidarius, Bombus lucorum*, and *Bombus pascuorum*) out of four species examined the majority of bumble bees circled in the same direction. Interestingly, the researchers did not observe any lateralization in *B. terrestris*. Since two species circled anticlockwise and one clockwise, it is unlikely that the asymmetry is a function of the structure of the florets.

Bumblebees observe and copy the behavior of others with regard to floral choices (Kawaguchi et al., 2007) and, moreover, they can learn to make nectar-robbing holes in flowers as a result of encountering them (Leadbeater and Chittka, 2008). Recently, Goulson et al. (2013) investigated handedness in nectar-robbing bumblebees (*Bombus wurflenii* and *Bombus lucorum*) feeding on *Rhinanthus minor*, a flower that can be robbed from either the right-hand side or the left-hand side and they looked at a possible effect of social learning on handedness. Numerous patches of *R. minor* spread across an alpine landscape were studied and each patch was found to be robbed on either the right or the left. The intensity of side bias increased through the season and was strongest in the most heavily robbed patches. Bees within patches seemed to learn robbing strategies (including handedness) from one another, either by direct observation or from experience with the location of holes, leading to rapid frequency-dependent selection for a common strategy, i.e., adopting the same handedness within particular flower patches.

Frasnelli et al. (2011) studied primitive social bees, stingless bees (Fam. Apidae, Tribe Meliponini – **Figure 1**) to shed light on the possible evolutionary origins of the left-right antennal asymmetry. Three species of Australian native, stingless bees (*Trigona carbonaria*, *Trigona hockingsi,* and *Austroplebeia australis*) were trained to discriminate two odors, lemon(+)/vanilla(-), using the PER paradigm. Recall of the olfactory memory at 1 h after training was better when the odor was presented to the right than to the left side of the bees. In contrast, recall at 5 h after training was better when the odor was presented to the left than to the right side of the bees. Hence, stingless bees (Meliponini) have the same laterality as honeybees (Apini), which may suggest that olfactory lateralization is likely to evolved prior to the evolutionary divergence of these species. The distributional pattern and fossil records are indicative of greater antiquity for the Meliponini compared to Apidi, Bombini, and Euglossini, and suggestive of an independent origin or an early divergence from a proto-other Apidae branch (Camargo and Pedro, 1992). However, the phylogenetic relationships among the four tribes of bees (i.e., corbiculate Apidae: Euglossini, Bombini, Meliponini, and Apini) are controversial and the single origin of eusociality is questionable. It has been suggested that eusociality evolved once in the common ancestor of the corbiculate Apidae, advanced eusociality evolved independently in the honeybee and in stingless bees, and that eusociality was lost in the orchid bees (Cardinal and Danforth, 2011). Considering this, it can be argued that the similarity found between honeybees and the three species of Australian stingless bees in population-level lateralization in recall of olfactory memory is linked with the social feature shared by the two tribes and may have evolved independently in the trajectory that led to honeybees and trajectory that led to stingless bees.

One can argue that the behavioral traits, such as olfactory learning and electroantennographic responsivity, investigated in the studies reported above (Rogers and Vallortigara, 2008; Anfora et al., 2010, 2011) are not obviously social in nature. However, it is not possible to exclude that the original drive for antennal asymmetries could be related to social interaction during for example trophallaxis, as observed in ants (Frasnelli et al., 2012b). Moreover, it is conceivable that some forms of asymmetries that are unlikely to have been directly selected as ESSs in social contexts could have evolved as population-level biases as by-product of other biases that in fact evolved as ESSs. It is likely that when an individual-level asymmetry is stabilized as a directional (population-level) asymmetry, other asymmetries that in principle would not require any alignment at the population level because they are irrelevant to any social interaction would organize themselves as directional as well simply because a directional organization in the two sides of the brain already exists.

Very recently Rogers et al. (2013b) investigated whether the rich social life of honeybees may be associated with directional biases in antennal use. Different social behavior (latency to contact, numbers of PER, number of C-responses, number of mandibulations) were analyzed in pairs of bees coming from either the same colony or from different colonies and having only their right antennae (left antennae removed) or only their left antenna (right antennae removed) or both antennae intact. The authors found a directional bias in the use of antennae for three measures of social interaction, latency, PER and C-responses. Dyads of bees tested using only their right antennae contacted after shorter latency and were significantly more likely to interact positively (proboscis extension) than were dyads of bees using only their left antennae. The latter were more likely to interact negatively (C-responses) even though they were from the same hive. In dyads from different hives C-responses were higher in dyads of bees using only their right antennae than in dyads of bees using only their left antennae. The right antenna seems, therefore, not only specialized for learning about new odors associated with food sources but also in exchange of odoriferous information between same-colony worker bees and in control of aggressive responses between different-colony worker bees. Use of the right antenna was also shown to motivate bees to approach and contact each other. In fact, although use of the left antenna did not cause bees to completely avoid each other, social behavior performed by the bees with only their left antennae intact was not context-appropriate, possibly due to an inability to distinguish between hive mates and bees from another hive. Hence, the right antenna seems to control social behavior appropriate to context, suggesting that lateral biases in behavior are associated with requirements of social life.

## LATERALIZATION IN VERTEBRATES AND INVERTEBRATES: COMMON ANCESTOR OR CONVERGENT EVOLUTION?

All the evidence about differences in the specializations of the left and right sides of the nervous system and behavior in invertebrates suggests that invertebrates share the attribute of lateralization with many vertebrates. This strengthens the conclusion that lateralization provides substantial advantages, since it has persisted, or evolved many times, in such diverse groups of animals. Asymmetries in invertebrates and vertebrates sometimes also show similarities in their appearance. One example is the processes of memory formation in parallel on the right and left sides of the brain and the interaction between the right and left memory traces during memory formation. In fruitflies, the transition from short- to long-term records of conditioning depends on an asymmetric body normally only present on the right side of the brain. When there is also a counterpart on the left, only short-term memory is formed (see The Fruit Fly *Drosophila melanogaster*). In honeybees, recall of short-term memory is possible through the right side, whereas recall of long-term memory is possible through the left side (see The Honeybee *Apis mellifera*). A shift of recall access from one to the other side of the brain has been observed previously in birds (Cipolla-Neto et al., 1982; Clayton, 1993; Andrew, 1999). This suggests that lateralized events in memory formation may be similar in arthropods and vertebrates and that the shifts from recently acquired information held independently by the right and the left sides to more integrated and complete long-term records should constitute a considerable advantage. Thus, because of this advantage mechanisms controlling such shifts have evolved (probably independently) in both arthropods and vertebrates.

The difficult and complex issue is whether homologous genes in invertebrates and vertebrates determined lateralization or whether there has been analogous evolution of lateralized function in the two taxa. It is probable that the common ancestor of metazoan animals specified the right-left axis (Vandenberg and Levin, 2009). Since it is also true of single-cell organisms such as ciliates, the same basic genetic mechanisms of specification of the left-right axis were probably present in the common ancestor of multi-cellular animals. The most striking evidence that the left-right axis may have been specified very early in metazoan evolution is the involvement of orthologs, i.e., homologous gene sequences in different species, of the Nodal family in the evolution of body plans and left-right specification in vertebrates (Boorman and Shimeld, 2002) and in Bilateria (Grande and Patel, 2009). The signaling molecule Nodal, a member of the transforming growth factor-β superfamily, is involved in the molecular pathway that leads to left-right asymmetry in vertebrates (Boorman and Shimeld, 2002) and in other deuterostomes, but no nodal ortholog had been reported previously in the two main clades of Bilateria: Ecdysozoa (including flies and nematodes) and Lophotrochozoa (including snails and annelids). Grande and Patel (2009) reported the first evidence for the presence of a nodal ortholog in a non-deuterostome group, indicating that the involvement of the Nodal pathway in left-right asymmetries might have been an ancestral feature of the Bilateria. Furthermore, this study suggests that nodal was present in the common ancestor of bilaterians and it too may have been expressed asymmetrically.

The recent comparison between the cellular and molecular mechanisms leading to neuronal asymmetries in the nematode *C. elegans* and in the zebrafish *Danio rerio* (Taylor et al., 2010) may also be helpful in the difficult and complex issue of the evolution of asymmetries in vertebrate and invertebrates. The specification of the left and right Amphid Wing “C” (AWC) neurons of the nematode olfactory system and the asymmetry in the fish epithalamus has been analyzed. It has been shown that both species use iterative cell–cell communication, i.e., reciprocal interactions between neural cells rather than a simple linear pathway, to establish left-right neuronal identity, and this reinforces the left-right asymmetry but with different outcomes and molecular details in each species. The functional differences in morphologically identical neurons in the olfactory system of *C. elegans* are the result of gap-junctional communication and calcium influxes, whereas the neuroanatomical left-right differences in the epithalamus of *D. rerio* are the result of morphogenic changes regulated by secreted signaling molecules. Thus, the invertebrate and vertebrate species considered share some commonalities in the mechanisms involved in asymmetrical neural development, i.e., the interaction of neurons across the midline during formation of the asymmetrical nervous system, and the inherently stochastic nature of some developmental pathways. However, results need to be interpreted with caution since the evolutionary gap between the 302 neurons of the worm and the estimated 78,000 neurons of the larval fish (Hill et al., 2003) is considerable. The striking differences in the genetic and cellular pathways underline the improbability that nematode and zebrafish lateralization arose from the same ancestral event. It is instead more reasonable to hypothesize that the left-right differences in the two species have evolved by convergence.

The ESS theory predicts that lateralization at the population-level is more likely to have evolved in “social” rather than in “solitary” species. Studies conducted in different species of insects seem to be in alignment with this prediction. Shoaling and not-shoaling fishes have also provided evidence in support of this hypothesis. In 20 species of teleost fishes, Bisazza et al. (2000) found that the shoaling ones (“social”) were lateralized for turning bias at the population-level; whereas the not shoaling ones were lateralized at the individual level but non at the population level (Bisazza et al., 2000; Vallortigara and Bisazza, 2002). Although lateralization in invertebrates may not be related to lateralization in vertebrates in an evolutionary sense, the social pressures associated with the need to coordinate asymmetric behaviors would hold irrespective of whether lateralization in vertebrates and invertebrates represent homology (common ancestor) or homoplasy (convergent evolution).

## Conflict of Interest Statement

The author declares that the research was conducted in the absence of any commercial or financial relationships that could be construed as a potential conflict of interest.
